# Aortic dissection following “ecstasy” use complicated by compartment syndrome

**DOI:** 10.1186/s12245-022-00461-1

**Published:** 2022-10-12

**Authors:** Erin McDonnell, Yi Zhou, Joshua Chao, Leonard Lee

**Affiliations:** 1 Rutgers Robert Wood Johnson Medical School, Piscataway, NJ 08854 USA; 2 JFK Johnson Rehabilitation Institute, Edison, NJ 08820 USA

**Keywords:** Acute aortic dissection, MDMA-induced aortic dissection, Compartment syndrome

## Abstract

**Background:**

Patients who present to the emergency department (ED) with acute chest pain should receive a thorough history and exam to rule out rare, life-threatening conditions, such as drug-induced acute aortic dissections (AD).

**Case presentation:**

A 34-year-old man with a history of uncontrolled hypertension, smoking, and “ecstasy” use presented to the ED with an acute type A aortic dissection (AD). Following surgery to repair the dissection, he developed compartment syndrome of the lower extremity requiring muscle excision and neurolysis with subsequent wound debridement procedures.

**Conclusion:**

Physicians treating adults with symptoms and signs of aortic dissection should take a focused history about substance use and include AD on their differential. In addition, the extremities should be monitored for signs and symptoms of ischemia throughout the acute peri-surgical period(s).

## Background

Patients who present to the emergency department (ED) with acute chest pain should be examined thoroughly to rule out rare, life-threatening conditions, such as acute aortic dissections (AD). 7% of patients who present with AD are under 40 years old and are less likely due to hypertension compared to older adults based on data from the International Registry of AD) [1]. We report a unique case about a patient who presented with a type A AD due to unmanaged hypertension and use of 3,4-methylenedioxymethamphetamine (MDMA — “ecstasy”), resulting in compartment syndrome of his lower extremity secondary to ischemia from the AD and subsequent malperfusion. Several case studies have described young patients who suffered from acute ADs a few hours after MDMA use, with one case resulting in gluteal compartment syndrome [2–5]. To our knowledge, this is the first patient reported in the literature to suffer from lower leg compartment syndrome secondary to MDMA-induced type A AD.

## Case presentation

A 34-year-old African American male presented to the emergency department (ED) due to sudden-onset severe chest pain. Thirty minutes prior to arrival, the patient experienced severe, tearing chest pain radiating to the back while smoking a cigarette. The patient reported shortness of breath and chest pain worsening with deep inspiration. He did not report any vision disturbances, headaches, or abdominal/leg pain. His past medical history was significant for current cigarette use (5 pack-years), 4 years of uncontrolled hypertension, and 5 years of chronic kidney disease (stage 3b). His home medications included amlodipine, clonidine, losartan, and metoprolol. The patient denied any diagnosis of connective tissue diseases such as Marfan or Ehlers-Danlos syndromes or Systemic Lupus Erythematosus. Family history was significant for type 2 diabetes in his mother and hypertension and kidney disease in his father who required dialysis and died in his 60s. Six months ago, the patient visited his primary care provider, admitting to non-adherence with antihypertensive medications. Five months prior, he began taking MDMA (“ecstasy”) three times a month, stating most recent ingestion was 2 days prior to hospitalization.

Upon arrival to the hospital, the patient was awake, oriented, afebrile, hypertensive (156/75 mmHg), and tachycardic (105 beats per minute). On auscultation, he was tachycardic with regular rhythm, with a holosystolic murmur. His lungs were clear to auscultation bilaterally. He had palpable, equal pulses, intact sensation, and 5/5 strength in all extremities. Labs demonstrated elevations in white blood cell count (24,200/μL), lactic acid (4.1 mmol/L), D-dimer (3150 ng/mL), blood urea nitrogen (24 mg/dL), and creatinine (2.5 mg/dL, baseline 3.0 mg/dL).

**Fig. 1:**
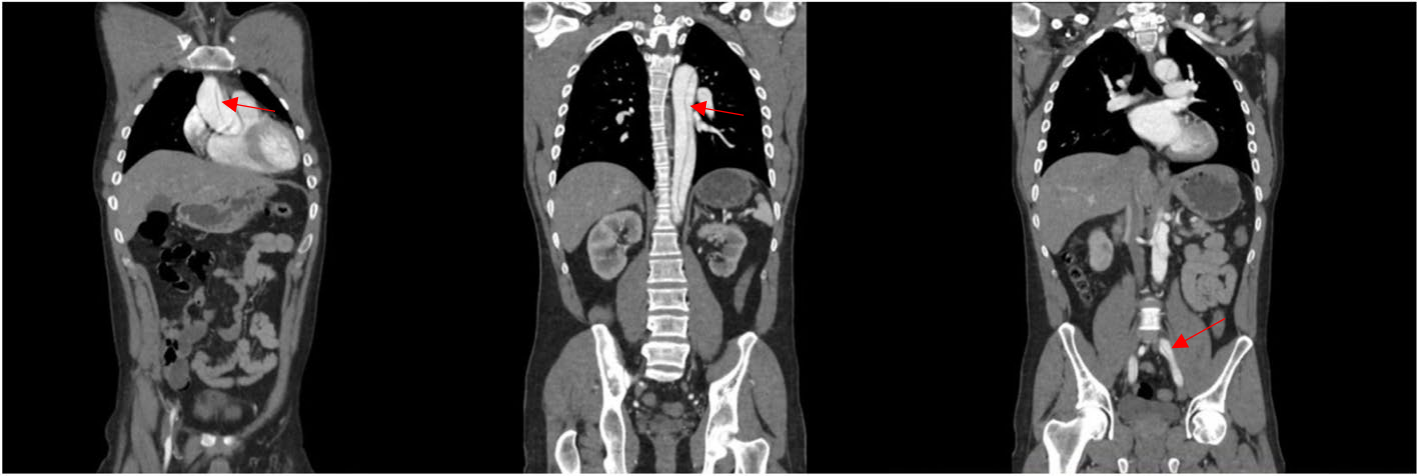
CT angiogram with contrast and coronal views depicting type A aortic dissection extending proximally to distally (left to right) to the iliac arteries and common femoral arteries bilaterally

SARS-CoV-2 (COVID-19) RT-PCR test was negative, and the patient denied recent symptoms consistent with COVID-19 infection. Electrocardiogram showed sinus rhythm with nonspecific ST changes, left ventricular hypertrophy, T-wave inversion in inferior/lateral leads, and questionable ST elevations in leads V1-V4. Computed tomography angiography (CTA) revealed a type A aortic dissection extending from the aortic root involving the ascending aorta, arch, descending thoracic aorta, and extending into the iliac and common femoral arteries (Fig. 1).

Upon hospital admission, the patient was started on a nicardipine drip with labetalol pushes for goal systolic blood pressure under 120 mmHg, and cardiac and vascular surgery were consulted for emergent surgery. The patient underwent median sternotomy, ascending aortic aneurysm and dissection repair with a gelatin-sealed, woven polyester thoracic endovascular stent graft to treat the descending thoracic aorta, aortic valve resuspension, right axillary artery, and right femoral venous cut down for cardiopulmonary bypass and intraoperative transesophageal echocardiogram.

Two days later, the patient had an acute worsening of hypertension and reported right lower limb pain, right calf swelling, and decreased function. On exam, the right leg had detectable pedal pulses but was tense and tender with pain on passive range of motion along with decreased strength on dorsiflexion and minimal plantarflexion. Labs revealed worsening creatinine to 5.4 (2.5 on admission) with rhabdomyolysis and a creatinine phosphokinase level of 1,105 U/L. Venous Dopplers were negative.

Urgent right lower limb 4-compartment fasciotomy for compartment syndrome was performed. Plastic surgery was consulted for evaluation and management of necrotic muscle and skin in the lateral compartment. Following evaluation, the patient underwent excision of peroneus longus and brevis muscles, lateral gastrocnemius, lateral hemisoleus and partial excision of flexor hallucis longus, and neurolysis of right common peroneal and superficial peroneal nerves. Following muscle excision, he underwent a debridement by plastic surgery, and wound vacuum-assisted closure (VAC) was applied.

The remainder of his hospital course was complicated by worsening acute kidney injury requiring dialysis. He underwent debridement two more times. The patient’s renal function continued to improve with good urine output. He was discharged to acute rehab with VAC to the medial and lateral compartments with dressing changes every 3 days with potential plan for skin graft in 2 to 4 months. He participated in acute rehabilitation for 10 days with continued wound VAC changes. Strict blood pressure control was recommended, and the patient was discharged home at an independent level with adaptive devices, including a rolling walker and straight cane.

## Discussion

We report a unique case which presents a young patient who suffered from compartment syndrome secondary to malperfusion from a type A AD extending into the femoral vessels. Typically, patients present with a sudden tearing chest pain radiating to the back, but patients can also present with syncope, stroke-like symptoms, and/or shortness of breath [1]. His past medical history of uncontrolled hypertension, smoking, and MDMA use possibly predisposed him to a weakening in the intimal layer of the aorta compared to other risk factors such as underlying connective tissue diseases or valvular abnormalities. The existing literature highlights only a few young patients who suffered from acute ADs due a few hours after MDMA use, with one case resulting in gluteal compartment syndrome [2–5]. Our patient’s MDMA ingestion prior to the event likely worsened his existing underlying hypertension secondary to his chronic kidney disease [6]. Our patient is unique in that he possibly suffered from lower leg compartment syndrome secondary to a MDMA-induced type A AD. Unlike reported cases of patients who presented with abdominal pain or lower limb pain during the acute AD, our patient did not describe any leg pain until after his aortic repair [7]. It was concluded that the patient’s compartment syndrome was secondary to reperfusion injury, possibly related to the AD. Intimal flap or clot was initially part of the differential but were excluded due to lack of evidence.

## Conclusion

Clinicians should closely monitor for signs of limb ischemia in a timely manner to preserve muscle in the setting of acute AD. Additionally, our patient serves as a reminder for clinicians to take detailed histories, including drug use, when interacting with patients who present with sudden-onset chest pain who are younger than the typical age of acute ADs.

## Data Availability

Data sharing is not applicable to this article as no datasets were generated or analyzed during the current study.

## Abbreviations

AD: Aortic dissection
MDMA: 3,4-Methylenedioxymethamphetamine
CTA: Computed tomography angiography
VAC: Vacuum-assisted closure

## References

[1] Januzzi JL, Isselbacher EM, Fattori R, Cooper JV, Smith DE, Fang J, Eagle KA, Mehta RH, Nienaber CA, Pape LA (2004). Characterizing the young patient with aortic dissection: results from the International Registry of Aortic Dissection (IRAD). J Am College Cardiol.

[2] Duflou J, Mark A (2000). Aortic dissection after ingestion of “ecstasy”(MDMA). Am J Forensic Med Pathol.

[3] Kanahara S, El-Refai M, Lakkis N, Tabbaa R (2016). Acute ascending aortic dissection after MDMA/ecstasy use: a case report. Hellenic J Cardiol.

[4] Ferrie R, Loveland RC (2000). Bilateral gluteal compartment syndrome after ‘ecstasy’hyperpyrexia. J Royal Soc Med.

[5] Swalwell CI, Davis GG (1999). Methamphetamine as a risk factor for acute aortic dissection. J Forensic Sci.

[6] Hall AP, Henry JA (2006). Acute toxic effects of ‘ecstasy’(MDMA) and related compounds: overview of pathophysiology and clinical management. Br J Anaesth.

[7] Yin Z, Yang JR, Wei YS, Liang BL, Wei YB, Zhou KQ, Wang Z, Yan B, Gao YL (2015). Ischemia-reperfusion injury in an aortic dissection patient. Am J Emerg Med.

